# Targeting NEAT1 Affects the Sensitivity to PARPi in Serous Ovarian Cancer by Regulating the Homologous Recombination Repair Pathway

**DOI:** 10.7150/jca.91896

**Published:** 2024-01-20

**Authors:** Yang Liu, Guoyan Liu

**Affiliations:** 1 Departments of Obstetrics and Gynecology, Tianjin Medical University General Hospital, Tianjin, 300052, China.; 2 Correspondence to: Dr. Guoyan Liu, Key Laboratory of Cancer Prevention and Therapy of Tianjin, Department of Gynecologic Oncology, Tianjin Medical University Cancer Institute and Hospital, National Clinical Research Center for Cancer, Tianjin's Clinical Research Center for Cancer, Huanhuxi Road, Hexi District, Tianjin, 300060, China.

**Keywords:** NEAT1, PARP inhibitor, Serous ovarian cancer, Homologous recombination repair, DNA damage

## Abstract

**Objective:** Patients initially sensitive to PARPi (PARP inhibitor) regain resistance because of homologous recombination (HR) restoration, although PARPi has a synthetic lethality effect on serous ovarian cancer cells with BRCA1/2 mutations. This study aimed to investigate the role of NEAT1 in HR function and whether targeting NEAT1 in serous ovarian cancer cells could increase PARPi sensitivity.

**Methods:** Ovarian cancer patients' clinical information and the expression of NEAT1 were collected from The Cancer Genome Atlas (TCGA) and the International Cancer Genome Consortium (ICGC). Ovarian cancer (OC) cells HeyA8 and SKOV3 were silenced by transfecting NEAT1 ASO. QRT-PCR confirmed the mRNA expression of RAD51, FOXM1, NEAT1_1 and NEAT1_2. We assessed the expression of RAD51, FOXM1, and γ-H2AX by Western blotting and Immunofluorescence. Comet Assays were used to detect DNA double-strand damage levels. In OC cells transfected with NEAT1 ASO or co-transfected overexpression RAD51/empty vector and si-NEAT1/si-ctrl, the sensitivity to Olaparib was determined using CCK8 assay. The Kaplan-Meier survival curves assessed the prognostic and predictive roles of NEAT1 in OC.

**Results:** NEAT1 was an independent prognostic marker of ovarian cancer. NEAT1 knockdown reduced the expression of NEAT1_1, NEAT1_2, RAD51, and FOXM1 and increased the expression of γ-H2AX. In addition, Olaparib increased the expression of RAD51, representing HR repair efficiency, which was inhibited by NEAT1 knockdown. Moreover, the knockdown of NEAT1 increased the DNA damage caused by Olaparib, demonstrated by increased nuclear γ-H2AX foci, DNA in the tail, and expression of γ-H2AX. NEAT1 knockdown sensitized ovarian cancer cells to Olaparib by targeting RAD51-HR. NEAT1 expression could predict response to chemotherapy for ovarian cancer.

**Conclusions:** NEAT1 knockdown inhibited HR capacity and increased DNA damage caused by Olaparib in serous ovarian cancer cells, making them more sensitive to Olaparib and providing a crucial therapeutic advantage of increasing sensitivity to Olaparib.

## Introduction

The most lethal gynecologic malignancy among female patients, ovarian cancer is the eighth leading cause of cancer-related deaths around the world. Currently, Olaparib is a promising targeted drug as first-line maintenance monotherapy for ovarian cancer, which had a BRCA1/2 mutation 1 and maintenance treatment of platinum-sensitive recurrent ovarian cancer 2. However, many patients had a poor response to PARPi because of acquired PARPi resistance 3-5, and the mechanisms of PARPi-acquired resistance remain to be deeply studied. Hence, how to target HR to develop “HRDness” (homologous recombination deficiency) and optimize the combination strategy to maintain the efficacy of PARPi is still a question worthy of further discussion.

The HR pathway is an important pathway to repair double-strand breaks (DSBs) using sister chromatids as templates during the S and G2 phases of the cell cycle 6. RAD51, one of the most important enzymes of the double-stranded DNA damage repair mechanism mediated by HR, replaces replication protein A (RPA) on the damaged DNA single-strand by binding with BRCA2 and catalyzes the exchange of homologous DNA sequences 7. RAD51 could be used to improve the efficacy of the DSBs-inducing agent etoposide (VP16) in small-cell lung cancer (SCLC) 8. Therefore, RAD51 is essential for HR function and tumor survival following DNA damage. FOXM1 regulates gene expression in HR and adaptive response to cellular oncogenic stress. Olaparib has been found to increase FOXM1 expression as an adaptive cellular response, and FOXM1 inhibitor could produce “BRCAness” to increase sensitivity to Olaparib. It indicated that targeting the FOXM1 pathway might prevent acquired resistance to PARPi 9.

LncRNA, a noncoding RNA of more than 200 nucleotides in length, has been associated with multiple cellular functions. The nuclear paraspeckle assembly transcript 1 (NEAT1) is an abundant intranuclear lncRNA that contains two transcript isoforms, NEAT1_1 (3.7 kb) and NEAT1_2 (23 kb). Also, NEAT1 regulates tumor cell proliferation, invasiveness, metastasis, and chemoresistance by acting as a miRNA sponge 10. As a result of the knockdown of NEAT1 and NEAT1_2, cancer cells MCF-7 were more responsive to the potent PARP inhibitor ABT-888 11. In human myeloma cell lines (HMCLs), they found NEAT1 silencing treatment increased anti-multiple myeloma drugs sensitivity, which indicated that a combination of NEAT1 silencing with Olaparib, bortezomib, carfilzomib, and melphalan showed a synergistic effect 12. These results suggested that targeting NEAT1 could be a promising therapeutic strategy to improve the efficacy of chemotherapeutic drugs. However, it has not been reported that NEAT1 played a role in regulating sensitivity to Olaparib in OC patients. Therefore, an in-depth study of the mechanism of NEAT1 in tumor drug resistance is of great significance in developing new treatment strategies to improve the sensitivity of ovarian cancer patients to chemotherapy.

Our previous research found that miR-506-3p overexpression played an essential role in regulating the sensitivity to cisplatin and Olaparib by inhibiting the CDK4/6-FOXM1 pathway and RAD51-HR axis in serous ovarian cancer cells 13, 14. NEAT1 functioned as competing endogenous RNA (ceRNA) for miR-506 by luciferase reporter assay and gene expression profile data in high-grade serous ovarian carcinoma (HGSOC) 15. In this research, NEAT1 knockdown could increase the extent of Olaparib on DNA damage, inhibit HR capacity raised by Olaparib, and increase sensitivity to Olaparib by inhibiting RAD51-HR and increasing DNA damage levels in serous cancer cells by sponging miR-506-3p. Therefore, we proposed that NEAT1 could be a potential therapeutic target for increasing Olaparib sensitivity in OC cells.

## Materials and methods

### Cell lines and cell culture

We get human ovarian cancer cell lines (SKOV3 and HeyA8) from the American Type Culture Collection (ATCC, Manassas, VA, USA). Both cell lines were cultured in RPMI-1640 medium (Pricella; PM150110) containing 10% fetal bovine serum (FBS) (Biological Industries; 04-001-1ACS), 100 IU of penicillin, and streptomycin (100X) (Pricella; PB180120).

### Reagents, Antibodies, and Plasmids

Antibodies we used are as follows: Secondary antibodies: goat anti‐rabbit (ZB-2301) and rabbit anti‐mouse (ZB-2305) secondary antibodies, Alexa Fluor 594-labeled goat anti-mouse (ZF-0513) and Alexa Fluor 488-labeled goat anti-rabbit antibodies (ZF-0511) were purchased from ZSGB-BIO. Anti-RAD51 antibody (Abcam; ab133534), FOXM1 (D12D5) XP® Rabbit mAb (Cell Signaling; #5436), Anti-gammaH2A.X (phospho S139) antibody (Abcam; ab26350), Mouse Anti-βactin mAb (ZSGB-BIO; TA-09).

Pharmacological agents: Olaparib (MedChemExpress; HY-10162), DMSO (Solarbio; D8371).

Plasmid DNA was isolated by plasmid DNA extraction kits (CWBIO; CW2105S) using the alkaline lysis method.

### Small interfering RNA-mediated knockdown of NEAT1

We silenced the expression of NEAT1_1 and NEAT1_2 using locked nucleic acid-modified antisense oligonucleotides (ASOs). NEAT1 ASO and its negative control interference sequences were conducted by RiboBio (Guangzhou, China). The interference sequences of NEAT1 ASO and negative control are listed as follows: ASO-NEAT1-004 (5'-AGGGACCACTTAAGACGAGA-3'), ASO-NEAT1-006 (5'-GGTCTGAGGAGTGATGTGGA-3'), ASO-NEAT1-009 (5'- GGGATGATGCAAACAATTAC-3') and negative control ASO-NC (5'- CCTTCCCTGAAGGTTCCTCC -3').

### Plasmid and siRNA transfection

Plasmid and ASO transfection were carried out by Lipofectamine 2000 transfection reagent (11668500) purchased from Invitrogen.

### RNA analysis and Real-time quantitative PCR

Following the provider's instructions, total cell RNA was extracted from both OC cells using TRIzol Reagent (GLPBIO; GK20008). RNA samples were stored at -20℃. 500 ng of RNA was reversed transcribed in a final volume of 20uL for the cDNA synthesis using cDNA Synthesis SuperMix kits (TransGen Biotech; AT341). The mRNA amounts of target genes were calculated using the 2^-ΔΔCT method and normalized according to the control condition.

### Immunofluorescence analysis

HeyA8 and SKOV3 cells were transiently transfected with si-NEAT1/si-ctrl for 48 hours, treated with Olaparib/DMSO for 48 hours, and treated with Olaparib/DMSO for 48 hours after transiently transfected with si-NEAT1/si-ctrl for 24 hours. Cells were fixed with 4% paraformaldehyde. After a rupture of membranes in 1X PBS containing 0.2% Triton-X 100 and blocking for one h with 5% goat serum in 1% BSA/PBS, primary antibodies (RAD51 and γ-H2AX) diluted in blocking solution were incubated for at least 12h at four °C. Cells were incubated with secondary antibodies diluted in a blocking solution the following day. Cells were then counter-stained with DAPI (40,6-diamidino-2-phenylindole) before mounting on glass coverslips. Images were captured with a laser confocal microscope (Zeiss, Oberkochen, Germany).

### Western blotting

The cell lysates were incubated in RIPA lysis buffer with protease inhibitors PMSF (100:1 ratio). The protein supernatant concentration was analyzed using a BCA kit (Solarbio; PC0020), and lysate proteins were boiled with a 5X loading buffer for 10 min. The denatured protein was resolved by 10-15% SDS-PAGE and transferred to PVDF membranes through western blotting. After that, the membrane was blocked using BSA (5% in TBST) and incubated overnight with antibodies against β-actin (1:2500), RAD51 (1:800), FOXM1 (1:500), γ-H2AX (1:500). After that, the membrane was washed with TBST and incubated with secondary antibody (1:5000). ECL reagent was used to develop blots, and imaging was captured using EasySee® Western Blot Kit (TransGen Biotech; DW101-01).

### Comet Assays

HeyA8 and SKOV3 cells were transfected with si-NEAT1/si-ctrl. Twenty-four hours later, NEAT1 knockdown cells were added with 75 μM Olaparib for another twenty-four hours. All treated cells were analyzed by single-cell gel electrophoresis. We used “comet tail DNA moment” to express the DNA strand breakage extent. Tail DNA content was analyzed with the Comet Assay Software Project (CASP Lab). We measured at least 100 cells per sample and calculated average damage from 3 independent experiments.

### *In-vitro* cell viability assays

We measured cell viability by the CCK8 assay kit (GLPBIO; GK10001). Briefly, 5000 cells/well were seeded on 96-well plates. After attachment for 24h, cells were incubated in a 1640 medium containing different concentrations of Olaparib for 48h. The fresh 1640 medium containing 10% CCK8 reagent was added per well, and OD 450nm was measured. Cell viability rates were calculated using the specific formula [OD (treated group)-OD (blank)]/ [OD (untreated group)-OD (blank)].

### Data collection and analysis

The NEAT1 and RAD51 mRNA expression matrix in different ovarian cancer cell lines was obtained from the Broad Institute Cancer Cell Line Encyclopedia (CCLE) (https://portals.broadinstitute.org/ccle). RNA-sequencing expression profiles of NEAT1 were obtained from the TCGA dataset (https://portal.gdc.com) and GTEx (https://commonfund.nih.gov/GTEx). Furthermore, an analysis of TCGA was performed using the UALCAN portal (http://ualcan.path.uab.edu/analysis-prot.html). Statistical analyses were constructed by the R v4.0.3 software package ggplot2 (v3.3.3). P-value < 0.05 was considered statistically significant. The expression and corresponding clinical information for NEAT1 were downloaded from the TCGA and the ICGC dataset (https://dcc.icgc.org/releases/current/Projects). The R software package ggstatsplot realizes the two-gene correlation map. Spearman's correlation analysis describes the correlation among genes. Kaplan-Meier Plotter databases (http://kmplot.com/) were used for the prognostic role in OC patients. In addition, PanCanSurvPlot (https://smuonco.shinyapps.io/PanCanSurvPlot/, TCGA and GSE17260 for overall survival (OS), GSE17260 for progression-free survival (PFS) were used to predict chemotherapy response of NEAT1 in OC patients. Gene ontology (GO) (geneontology.org) and Kyoto Encyclopedia of Genes and Genomes (KEGG) (genome.jp/kegg) analyses of differentially expressed lncRNA NEAT1-associated genes were performed.

### Statistical analysis

All graphs and statistical results were presented using GraphPad Prism 8 software and SPSS. If continuous variables fit the normal distribution, we used the student's t-test to calculate the differences between the two groups. If not, we used non-parametric tests. All data are shown as the means ± SD at least three independent experiments with P value < 0.05 considered statistically significant.

## Results

### Expression analysis of NEAT1

**Figure 1:**
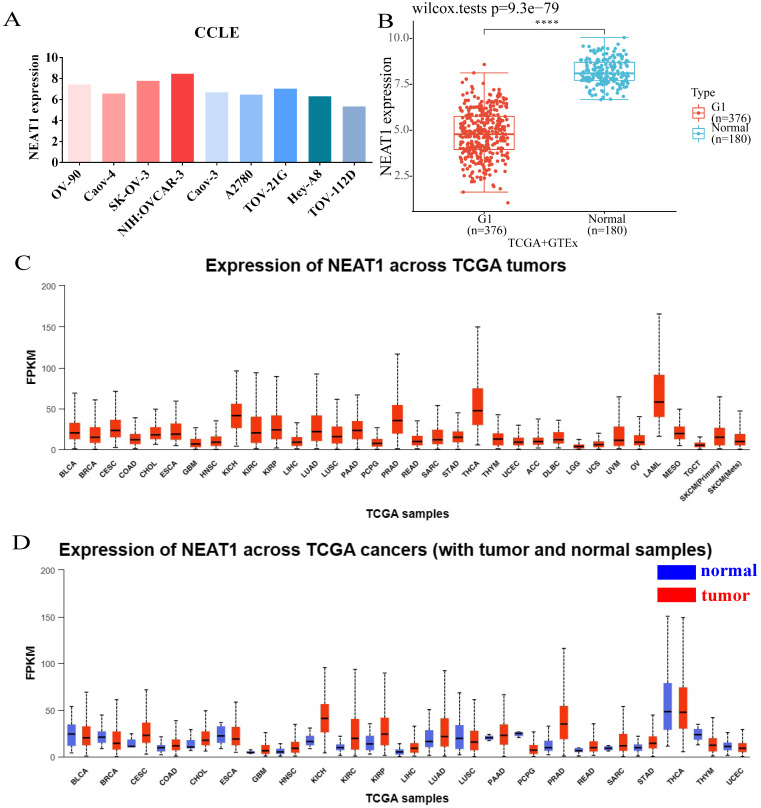
Expression of NEAT1 in ovarian cancer cells and among tumors. (FPKM: Fragments Per Kilobase Million; OV: ovarian serous cystadenocarcinoma) **(A)** CCLE databases showed the expression of NEAT1 in different ovarian cancer cell lines. **(B)** Differential expression analysis of NEAT1 between TCGA + GTEx database. **(C)** NEAT1 mRNA expression across TCGA tumors. **(D)** NEAT1 mRNA expression across TCGA tumors compared with normal samples.

We analyzed the expression level of NEAT1 in nine ovarian cancer cell lines using the online database CCLE. The expressions of NEAT1 in most ovarian cancer cell lines were relatively high (Figure 1A). We chose SKOV3 and HeyA8 cell lines in the follow-up experiment to finish *in vitro* detection. We utilized RNA-seq data from the TCGA database to analyze the expression of NEAT1 among various tumor tissues. NEAT1 was highly expressed in a wide variety of tumors compared to normal tissues (Figure 1C-1D). Unexpectedly, using data from the TCGA and GTEx, we found that NEAT1 expression was downregulated in ovarian cancer tissues compared to normal tissues (Figure 1B). We supposed that there are no normal ovary tissues in TCGA databases. However, studies have already focused on the expression of NEAT1 in OC. HGSOC tissues qPCR analysis showed that total NEAT1 and NEAT1-2 were expressed at significantly higher levels in HGSOC tissues than in normal ovarian tissues 15. Similarly, NEAT1 was upregulated in most ovarian tissues compared to para-tumor tissue specimens detected by qRT-PCR 16, 17.

### Enrichment analysis of NEAT1 in OC

**Figure 2:**
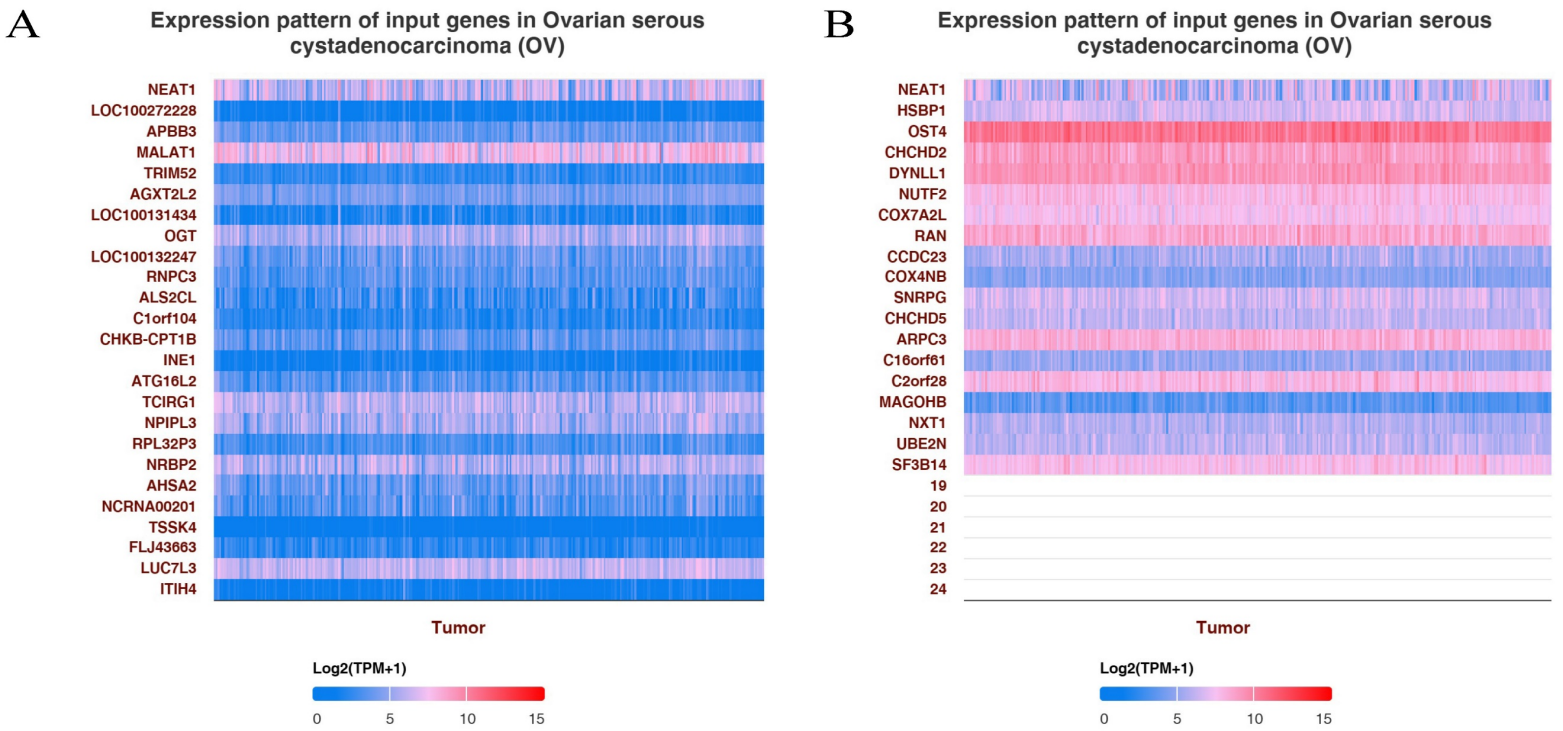
GO enrichment pathways of NEAT1 and its dysregulated genes in ovarian cancer patients. **(A)** The top 25 positive genes correlated with NEAT1 were displayed in a heatmap. **(B)** The top 25 negative genes correlated with NEAT1 were displayed in a heatmap.

**Figure 3:**
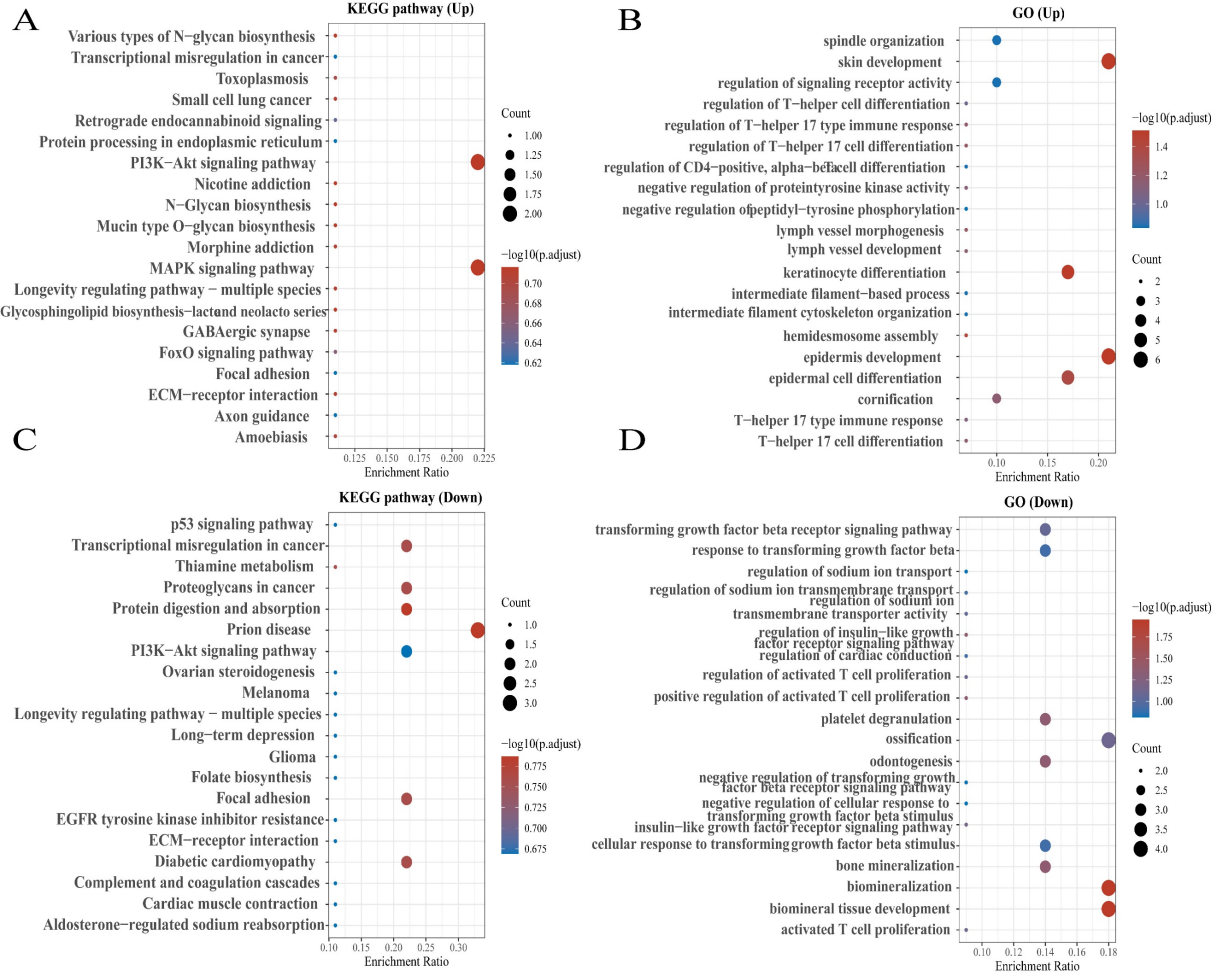
KEGG enrichment pathways of NEAT1 and its dysregulated genes in ovarian cancer patients. Colors represent the significance of differential enrichment, and the size of the circles represents the number of genes; the larger the circle, the greater the number of genes. The abscissa indicated gene ratio, and the enriched pathways were presented in the ordinate. **(A)** Bubble chart showing the top 20 KEGG pathways prediction of target genes of upregulated NEAT1. **(B)** GO enrichment analysis of upregulated NEAT1 symbols ranked by [-log10(P-value)] and number of genes. **(C)** The top 20 pathways of the target genes of downregulated NEAT1 were identified using KEGG analysis. **(D)** Top 20 generally changed GO terms of significantly downregulated NEAT1 symbols ranked by [-log10(P-value)] and number of genes.

To explore the functions and pathways affected by NEAT1 in OC, we identified genes positively or negatively co-expressed with NEAT1 using TCGA data by UALCAN analysis. We showed the top positive (Figure 2A) and negative (Figure 2B) 25 genes correlated with NEAT1 in a heatmap. Furthermore, we divided ovarian cancer patients into two groups (high and low) according to NEAT1 expression level using the ICGC database. We found 37 upregulated and 22 downregulated genes in the volcano plot (Figure S1). The enriched KEGG signaling pathways were selected to demonstrate the primary biological actions of NEAT1. GO analysis was used to seek potential targets of NEAT1. GO and KEGG pathway analysis showed 20 upregulated and downregulated GO and KEGG terms. The most significant GO terms were associated with “skin development” and “epidermis development” (Figure 3B, 3D). In addition, “PI3K-Akt signaling pathway” and “MAPK signaling pathway” were most closely associated with high NEAT1 expression in ovarian cancer (Figure 3A, 3C).

### Knockout of NEAT1 inhibited RAD51 and FOXM1 levels participated in HR

To further explore the expression pattern of NEAT1 and RAD51 in ovarian cancer, the online database CCLE was used to analyze the expression of NEAT1 in different ovarian cancer cell lines. The expressions of NEAT1 and RAD51 in ovarian cancer cells were negatively correlated (Figure S2A). We further analyzed the correlation between NEAT1 and DNA repair pathway scores in OC using the Spearman correlation using the R software GSVA package. We found that NEAT1 and DNA repair scores were negatively correlated (Figure S2B). However, we found a positive correlation between NEAT1 expression and HR-associated genes such as RAD50, RAD52, ATM, and ATR in OC (Figure S2C-F).

**Figure 4:**
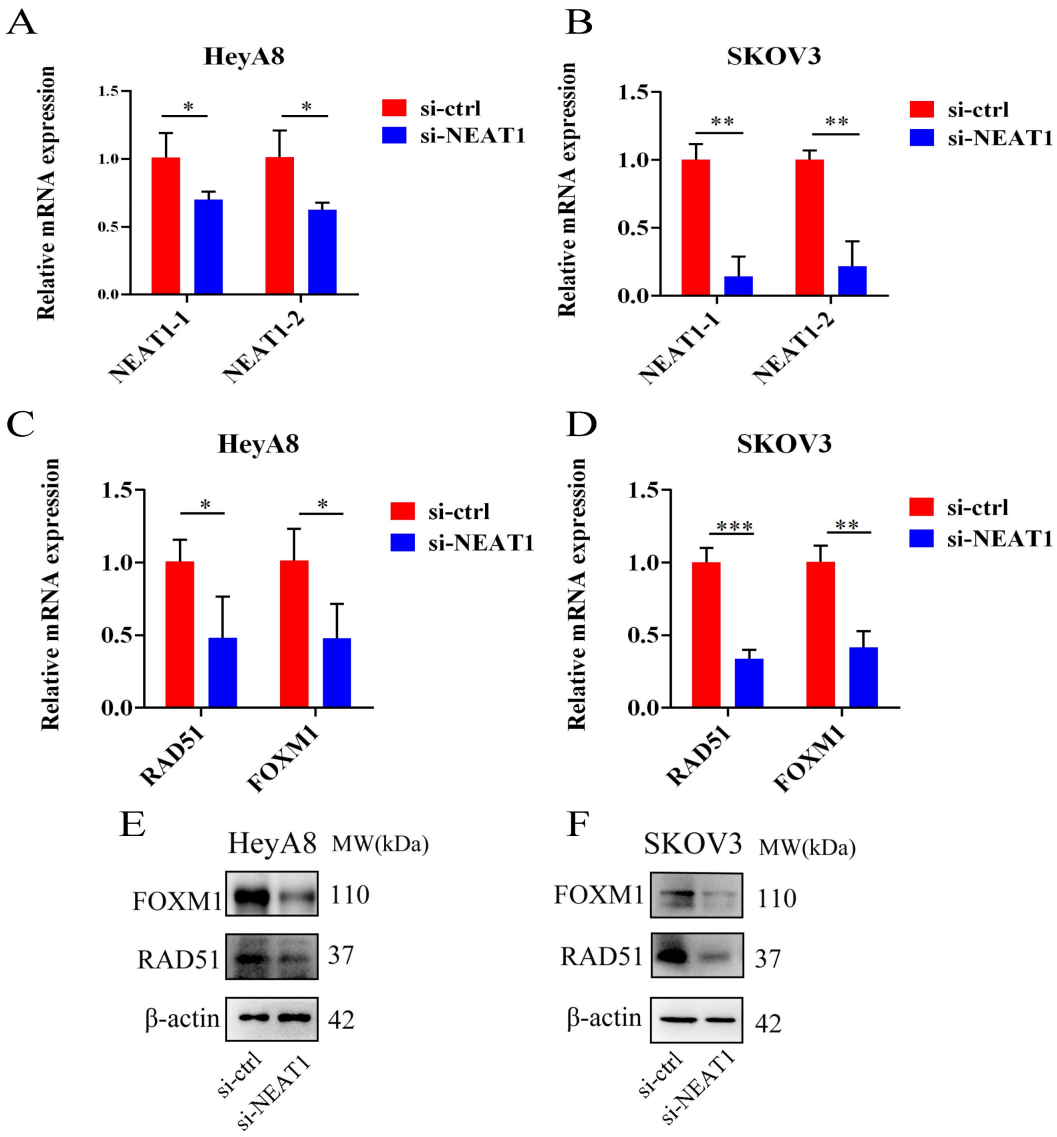
Effect of NEAT1 knockdown on homologous recombination repair. Data are shown as mean ± SD. ****P < 0.0001, ***P < 0.001, **P < 0.01, *P < 0.05. **(A and B)** NEAT1_1 and NEAT1_2 were detected in HeyA8 and SKOV3 by qRT-PCR (24h after transfection); NEAT1 expression was calculated as 2-ΔΔCt compared to negative control simultaneously. **(C and D)** Knockdown NEAT1 decreased RAD51 and FOXM1 mRNA levels in both cell lines, as confirmed by the qRT-PCR. **(E and F)** Both OC cell lines were transfected with si-NEAT1 compared to si-ctrl for 48h, and western blot assays detected RAD51 and FOXM1 protein expression.

We used three designed sequences (ASO-004 006 009) using ASO technology to obtain the highest levels of NEAT1 silencing. We verified the efficiency of NEAT1 silencing by qRT-PCR analysis after transfected with NEAT1 ASO compared to ASO-NC for 24h. HeyA8 NEAT1 RNA levels were significantly reduced upon NEAT1 ASO-004/si-NEAT1 treatment (Figure 4A). Similarly, SKOV3 NEAT1 RNA levels decreased upon NEAT1 ASO-006/si-NEAT1 treatment (Figure 4B).

RAD51 significantly affected HR response to DNA damage 18. In previous studies, it has been demonstrated that RAD51 knockdown reduced homologous recombination efficiency by homology-directed repair assay 13. Also, we found that miR-506-3p overexpression could increase the sensitivity to Olaparib or cisplatin by targeting RAD51 in ovarian cancer cells. Next, we proved that NEAT1 knockdown could regulate the expression of HR gene RAD51 by sponging miR-506-3p. Knockout of NEAT1 decreased RAD51, FOXM1 mRNA, and protein levels in both cell lines, which were confirmed by the qRT-PCR and western blot (Figure 4C-D, E-F). The results demonstrated that knockdown NEAT1 negatively regulated the expression of HR-associated genes RAD51 and FOXM1.

**Figure 5:**
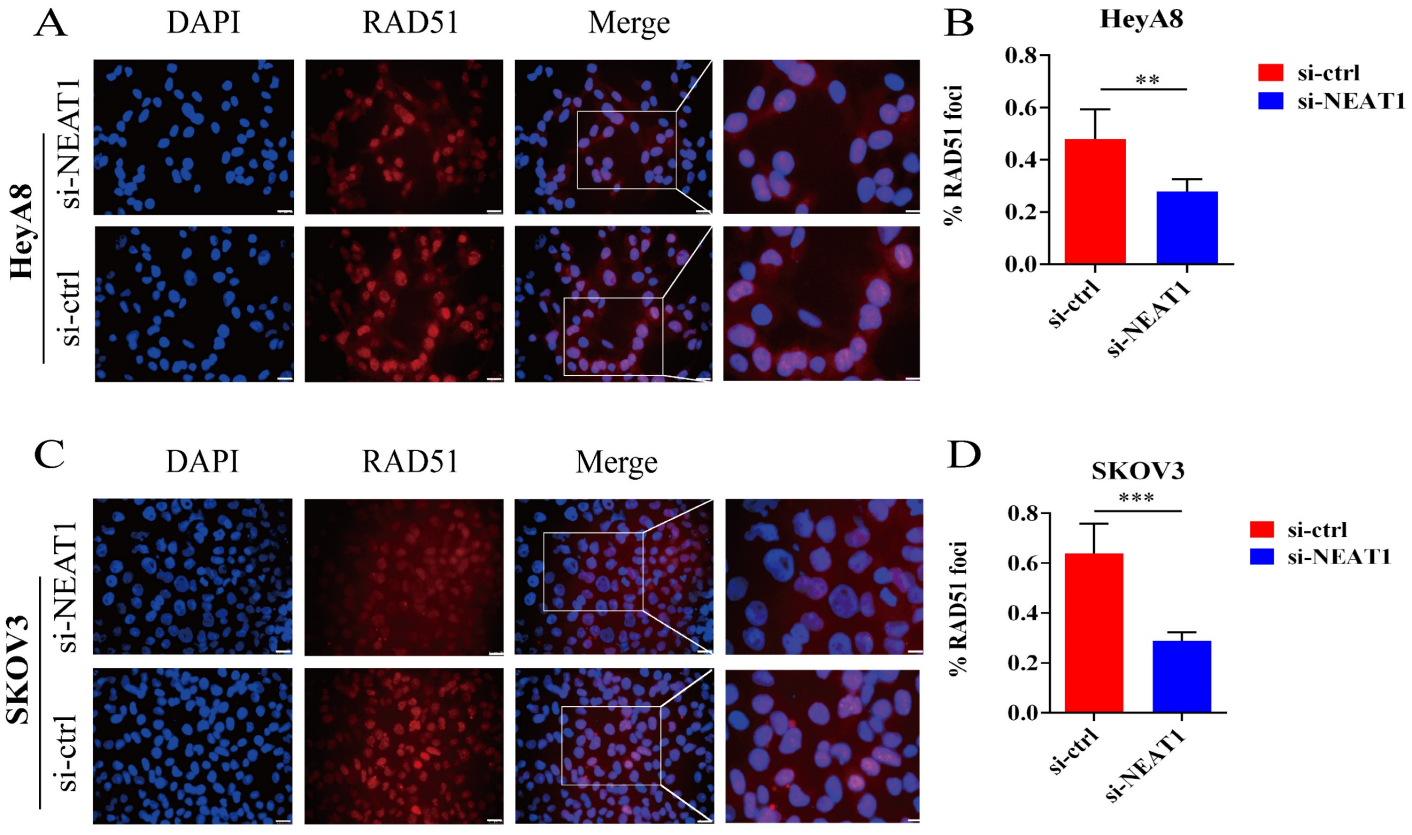
Effect of NEAT1 knockdown on RAD51 foci formation. Scale Bars: 25um and 100um (on the rightmost column). **(A and C)** Immunofluorescence focal microscopy examined the RAD51 foci formation of both OC cells transfected with si-NEAT1 for 48h. **(B and D)** The average ratio of RAD51 foci was collected in OC cells transfected with si-NEAT1/ctrl.

In addition, RAD51 is re-localized within the cell nucleus in response to cellular DNA damage, and the quantification of RAD51 nuclear foci could be seen as a promising marker of homologous recombination and PARPi resistance in g-BRCA tumors and HGSOC 19, 20. To further study whether NEAT1 regulates HR, we detected the capacity to form RAD51 foci after silencing NEAT1 by immunofluorescence staining analysis in both cell lines. We found that knockout of NEAT1 decreased RAD51 foci formation more than the control treatment group, indicating a defect in HR (Figure 5A-D).

### NEAT1 knockdown increased DNA damage

**Figure 6:**
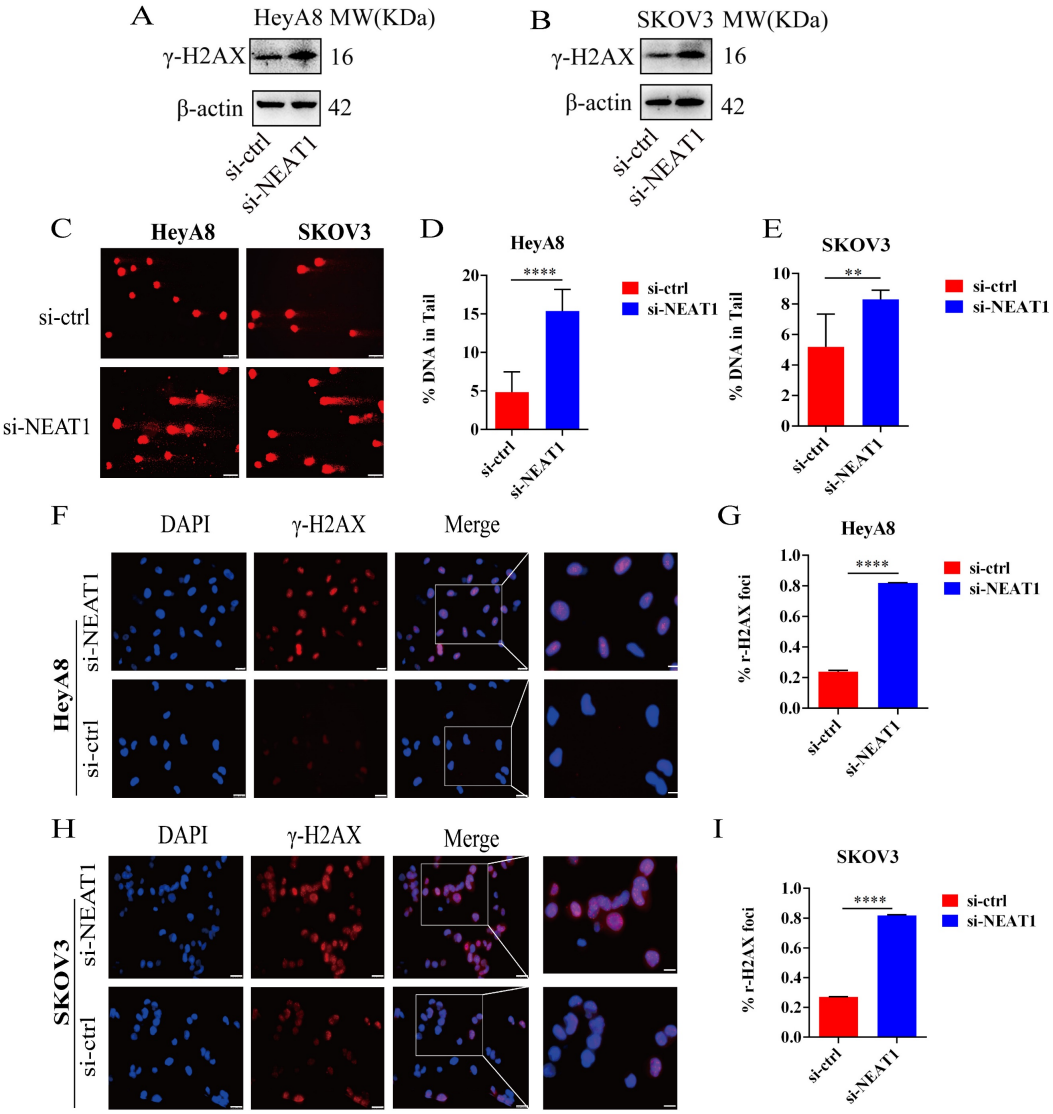
Effect of NEAT1 knockdown on DNA damage. **(A-B)** Western blot assays detected γ-H2AX protein expression after NEAT1 knockdown in HeyA8 and SKOV3 cells. **(C-E)** Unrepaired DNA breaks were measured by alkaline comet assay after knocking down NEAT1. Representative images are presented in the left panel, and the mean ± SD of tail DNA moment for each condition on the right. Scale Bars: 75um (C) **(F and G)** γ-H2AX foci were examined in HeyA8 after transfecting si-NEAT1 by immunofluorescence staining. The number of γ-H2AX foci was shown in cells transfected with si-NEAT1/ctrl. Scale Bars: 25um and 100um (F). **(H and I)** Similarly, γ-H2AX foci were detected in SKOV3 after transfecting si-NEAT1 or si-ctrl by immunofluorescence staining. Scale Bars: 25um and 100um (H).

To study whether the inhibition of HR induced by NEAT1 knockdown was relevant to increased DNA damage, we analyzed the protein expression and distribution of the γ-H2AX, a representative marker of DNA damage and repair. As shown in Figures 6A and 6B, NEAT1 knockdown improved the γ-H2AX expression shown by WB analysis. In addition, we measured DNA damage by alkaline comet assay. The results from the comet assay indicate that knockout of NEAT1 increased tail DNA moment and DNA damage more than control cells (Figure 6C-E). Moreover, we observed increased nuclear γ-H2AX foci, representing the site of DNA damage, in NEAT1 silencing OC cells by immunofluorescence staining (Figure 6F-G, H-I). We found that low NEAT1 expression and DNA replication pathway score was highly correlated (Figure S3A) using the TCGA dataset. In addition, the G2-M checkpoint score and common NEAT1 expression were positively related (Figure S3B). Together, these data suggested that the knockout of NEAT1 increased DNA damage levels.

### Olaparib induced the expression of HR repair genes

**Figure 7:**
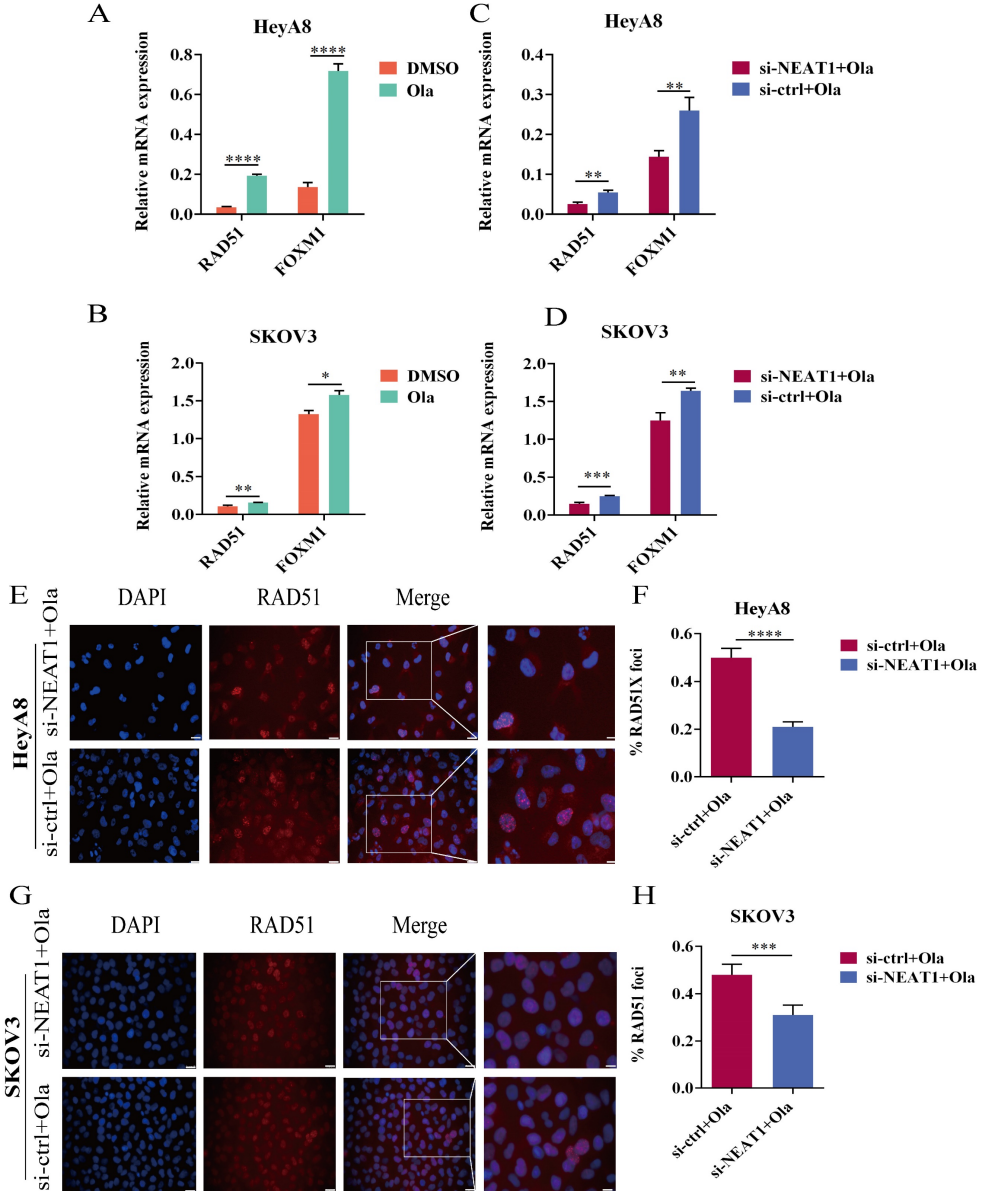
Olaparib-mediated regulation of RAD51, FOXM1, and NEAT1 knockdown inhibited the HR efficacy induced by Olaparib. Data are shown as mean ± SD. Ola: Olaparib. **(A and B)** RAD51 and FOXM1 mRNA levels were measured in two groups (Olaparib or DMSO treatment) in HeyA8 and SKOV3 cells. **(C and D)** RAD51 and FOXM1 mRNA levels were measured in two groups (transfected si-NEAT1 or si-ctrl combined with 75uM Olaparib treatment) in HeyA8 and SKOV3 cells. **(E and F)** Immunofluorescence focal microscopy detected RAD51 foci formation in si-NEAT1 or si-ctrl HeyA8 cells combined with Olaparib treatment. Scale Bars: 25um and 100um (E). **(G and H)** Similarly, immunofluorescence focal microscopy detected RAD51 foci formation in si-NEAT1 or si-ctrl SKOV3 cells combined with Olaparib treatment. Scale Bars: 25um and 100um (G).

In acquired PARPi-resistant cells and CCNE1 amplification/HR-proficient platinum-resistant cells, RAD51 nuclear foci formation significantly increased after PARPi treatment, indicating Olaparib-induced homologous recombination restoration 21. Furthermore, in BRCA gene wild-type ovarian cancer cell lines SKOV3 and OVCAR3, nuclear RAD51 foci could be induced by rucaparib, which might be decreased by CHK1 inhibitors 22. PARP inhibitors NU1025 caused RAD51 foci formation in the V-C8 (BRCA2-deficient) cell line, showing that PARP inhibitors treatment could increase γ-H2AX and RAD51 foci formation 23. Olaparib has been found to increase FOXM1 expression and the combination of FOXM1 and RAD51. Olaparib activated the FOXM1 pathway as an adaptive cellular response to DNA damage, and FOXM1 inhibition enhanced sensitivity to PARPi in ES-2 and OVCA420 ovarian cancer cells 9. We observed a significant improvement in RAD51 and FOXM1 mRNA expression in the Olaparib treatment group within 24 hours (Figure 7A-B).

### Knockout of NEAT1 inhibited the HR efficacy increased by Olaparib

We conducted qRT-PCR and western blot to explore whether silencing NEAT1 influenced the expression of HR repair genes, which Olaparib increased. We found that silencing NEAT1 significantly reduced the Olaparib-increased RAD51 expression. HeyA8 cells with NEAT1 knockdown combined with Olaparib treatment had statistically significantly lower mRNA levels of RAD51 and FOXM1 than control cells (Figure 7C). In addition, the qRT-PCR analysis showed a similar result in SKOV3 OC cells (Figure 7D).

To further discern whether silencing NEAT1 inhibited PARPi-induced DSB repair, we examined RAD51 foci formation. We found that Olaparib remarkably induced the formation of RAD51 nuclear foci while silencing NEAT1 significantly decreased Olaparib-induced RAD51 foci in OC cell lines (Figure 7E-F, 7G-H). These findings corroborated that silencing NEAT1 inhibited PARPi-induced HR repair in ovarian cancer cells.

### NEAT1 knockdown increased DNA double-strand break-induced by Olaparib

**Figure 8:**
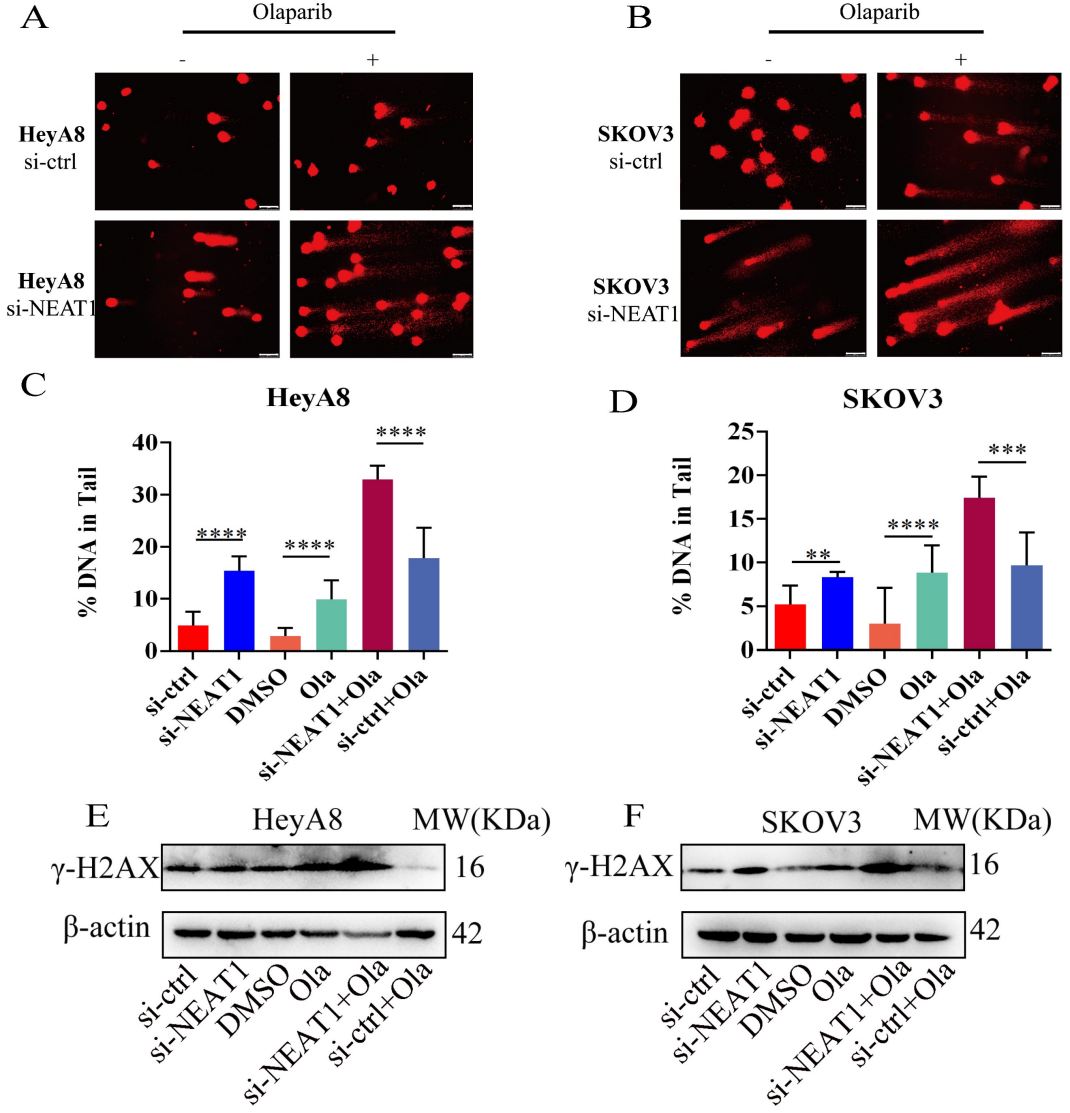
NEAT1 knockdown increased DNA double-strand breaks induced by Olaparib. Data are shown as mean ± SD. Scale Bars: 75um **(A and B)**. **(A and C)** Unrepaired DNA breaks were measured by alkaline comet assay after transfecting with si-NEAT1 or si-ctrl combined with Olaparib treatment in HeyA8 cells. Representative images are presented in the upper panel, and each condition's mean ± SD of the tail DNA moment is at the bottom. **(B and D)** DNA double-strand breaks were measured by comet assay after transfecting with si-NEAT1 or si-ctrl combined with Olaparib treatment in SKOV3 cells. Representative images are presented in the upper panel, and each condition's mean ± SD of the tail DNA moment is at the bottom. **(E and F)** γ-H2AX protein levels were examined in HeyA8 and SKOV3 cell lines under the above six conditions.

Next, we detected the extent of DNA damage by the comet assay. Knockout of NEAT1 combined with Olaparib markedly increased DNA extent in the tail, representing more severe DNA damage. We observed a statistically significant difference in the DNA content in the tail of HeyA8 cells (Figure 8A-B). Also, in SKOV3 cells, we observed a similar result (Figure 8C-D). Knockout of NEAT1 combined with Olaparib significantly increased γ-H2AX protein expression levels than Olaparib treatment alone (Figure 8E-F​).

**Figure 9:**
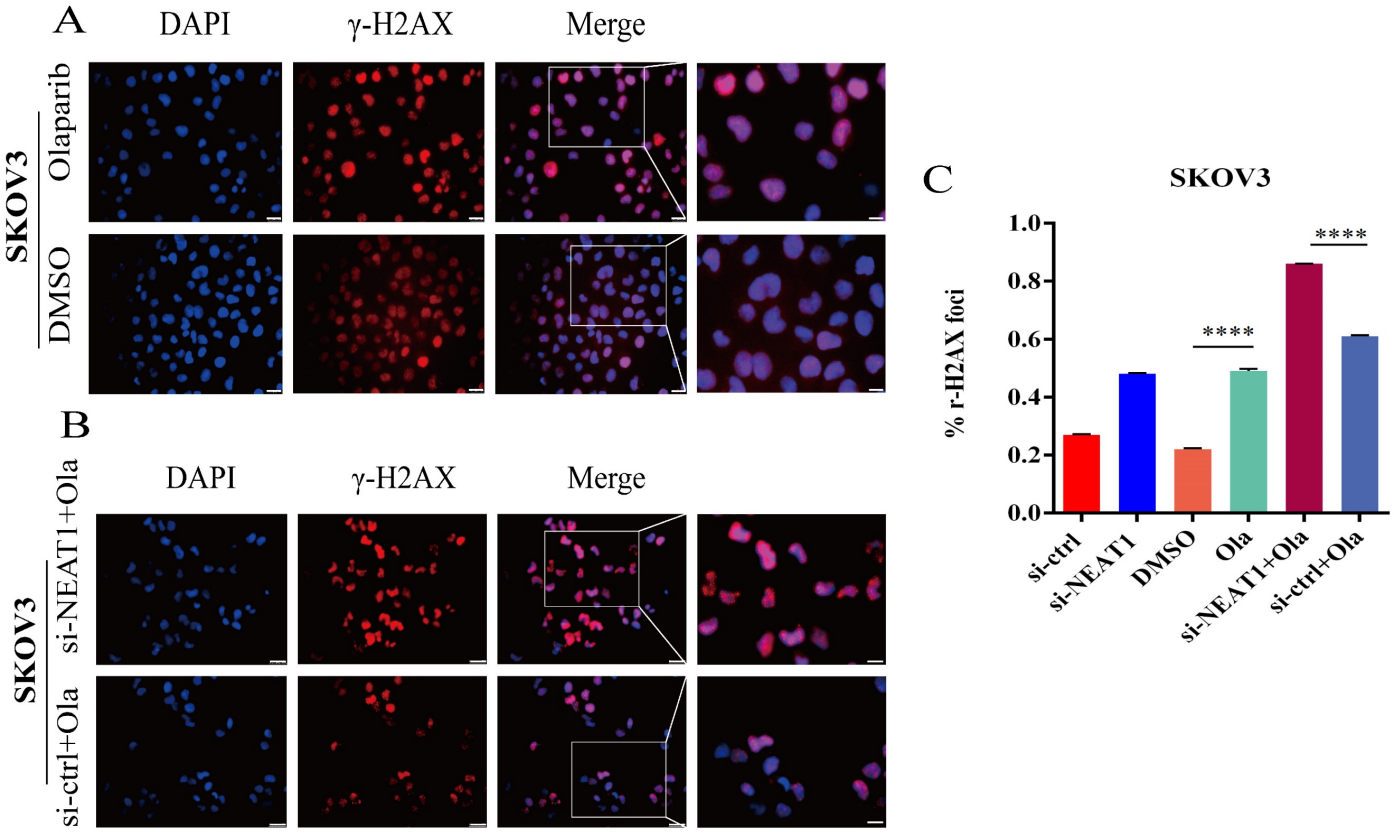
NEAT1 knockdown increased γ-H2AX foci formation induced by Olaparib. Data are shown as mean ± SD. Scale Bars: 25um and 100um (A and B). **(A)** Immunofluorescence focal microscopy detected γ-H2AX foci formation in SKOV3 cells after Olaparib or DMSO treatment. **(B)** Immunofluorescence focal microscopy detected γ-H2AX foci formation in SKOV3 cells after NEAT1 knockout or its control combined with Olaparib treatment. **(C)** The average ratio of γ-H2AX foci was examined in SKOV3 cells under the above six conditions.

To support the above results, we further found much more elevated γ-H2AX foci in SKOV3 cells treated with the combination of knockdown NEAT1 and Olaparib than cells treated with NEAT1 knockdown or Olaparib treatment alone by immunofluorescence assay (Figure 9A-C). These results demonstrated that silencing NEAT1 combined with Olaparib could cause a synergistic effect on DNA damage in OC.

### NEAT1 knockdown sensitized ovarian cancer cells to Olaparib

**Figure 10:**
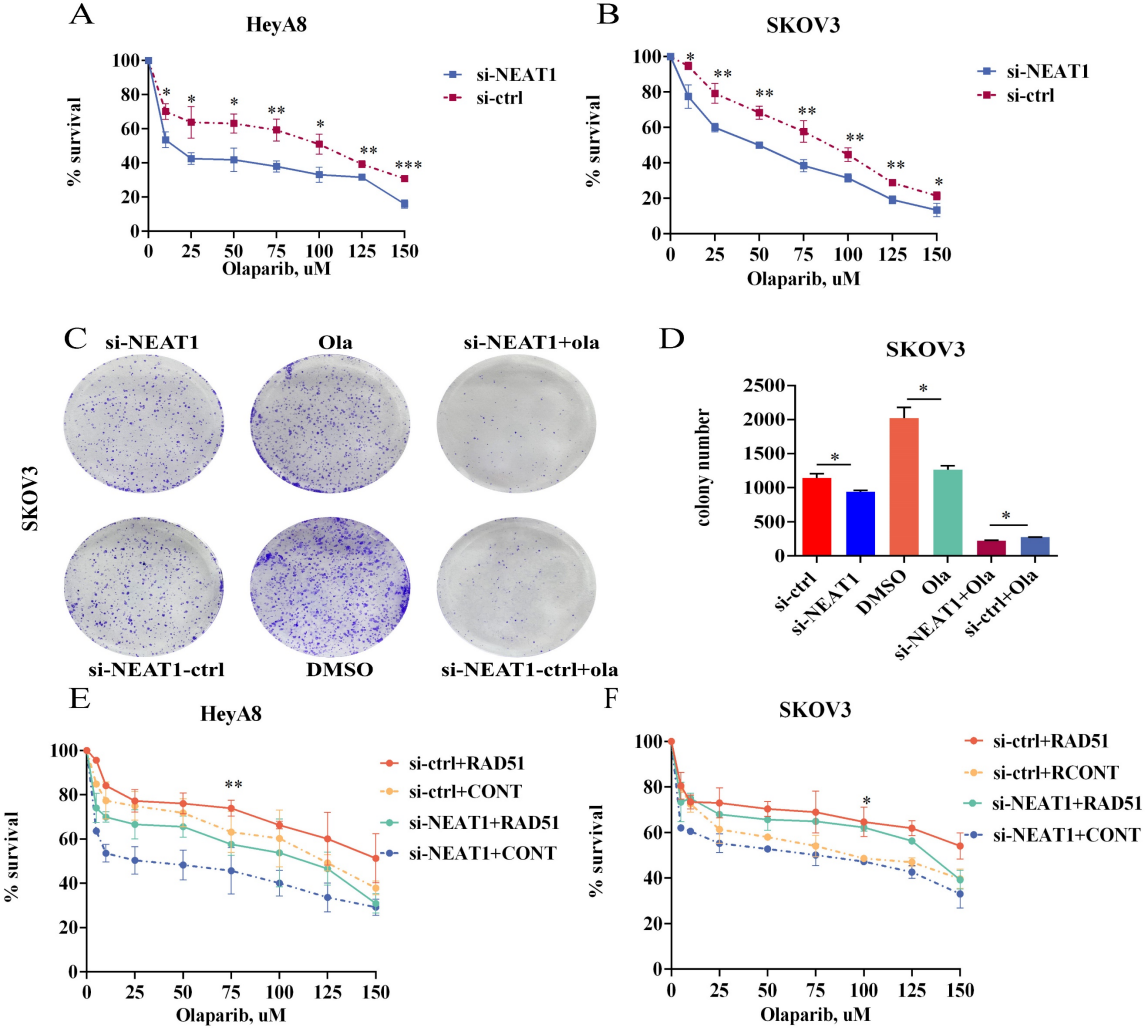
Targeting NEAT1 and RAD51 increased PARP inhibitor sensitivity in OC cells. Data are shown as mean ± SD. **(A-B)** HeyA8 and SKOV3 cells were transfected with 50 nM si-NEAT1 or si-ctrl. Cells were reseeded into 96-cell plates for Olaparib sensitivity assay. **(C-D)** 24h after transfecting si-NEAT1, Olaparib treatment, and NEAT1 knockout combined with Olaparib treatment, cells were reseeded for clonogenic cell-survival assay. **(E-F)** HeyA8 and SKOV3 cells were co-transfected with overexpression RAD51 or empty vector (EV) together with 50nM si-NEAT1 or si-ctrl. Cells were collected for Olaparib sensitivity assay 24 h later. Curves were generated from three independent experiments.

Because NEAT1 knockdown decreased RAD51 levels and HR ability, we wondered whether knockout of NEAT1 sensitized ovarian cancer cells to Olaparib. To explore the level to which NEAT1 expression contributes to Olaparib sensitivity in OC cells, we evaluated the occurrence of synergistic effects with Olaparib. We treated HeyA8 and SKOV3 with rising concentration (10-150 uM) of Olaparib in NEAT1 knockdown/negative control and then detected cell viability 48 h later. We observed a significant cell viability decrease through CCK8 assays in NEAT1 knockdown cells compared with the control group upon different concentrations of Olaparib (Figure 10A-B​). Also, NEAT1-induced sensitization to Olaparib was verified by a clonogenic survival assay. NEAT1 knockdown further decreased cell growth in Olaparib-treated cells, demonstrating a synergistic effect of the combination of NEAT1 silencing and chemotherapeutic drugs in ovarian cancer (Figure 10C-D​).

### RAD51 mediated the effects of NEAT1 on OC sensitivity to Olaparib

One of the PARPi resistance mechanisms is the recovery of RAD51 expression in HGSOC. We further explored whether RAD51 overexpression played an essential part in NEAT1 knockdown-induced cellular sensitivity to Olaparib. To further understand the mechanisms by which NEAT1 regulates the sensitivity to Olaparib in OC cells, we treated HeyA8 and SKOV3 with increased concentration of Olaparib after co-transfecting overexpression RAD51/empty vector and si-NEAT1/si-ctrl. Then, we calculated cell viability 48 hours later. First, we overexpressed RAD51 by transfecting the overexpressed RAD51 plasmid into OC cells, and qRT-PCR analysis showed that the RAD51 mRNA level increased significantly (Figure S4). Then, we found that the effect of knockdown NEAT1 on Olaparib sensitivity was partially rescued by overexpressing RAD51 (Figure 10E-F), suggesting that the knockdown of NEAT1-mediated sensitivity to Olaparib partly because of RAD51 expression suppression.

### Relationship between NEAT1 expression and clinicopathological features in OC

We contrasted NEAT1 expression in OC with clinical parameters, including age, race, tumor grade, and TP53 mutation status by UALCAN. We found high NEAT1 expression was associated with age (Age(41-60Yrs)-vs-Age(81-100Yrs): 0.089), tumor grade (Grade2-vs-Grade3: 0.0276), race (Caucasian-vs-Asian: 0.0893) (Figure S5). TP53 mutation status is obtained from TCGA whole exome sequencing data. The samples with/without TP53 mutation were matched with RNA-seq data. These results illustrated that high NEAT1 expression could increase the malignant degree of OC and result in a worse prognosis for OC patients, confirming that NEAT1 might be an oncogene of OC.

### NEAT1 as a prognostic predictor in OC

**Figure 11:**
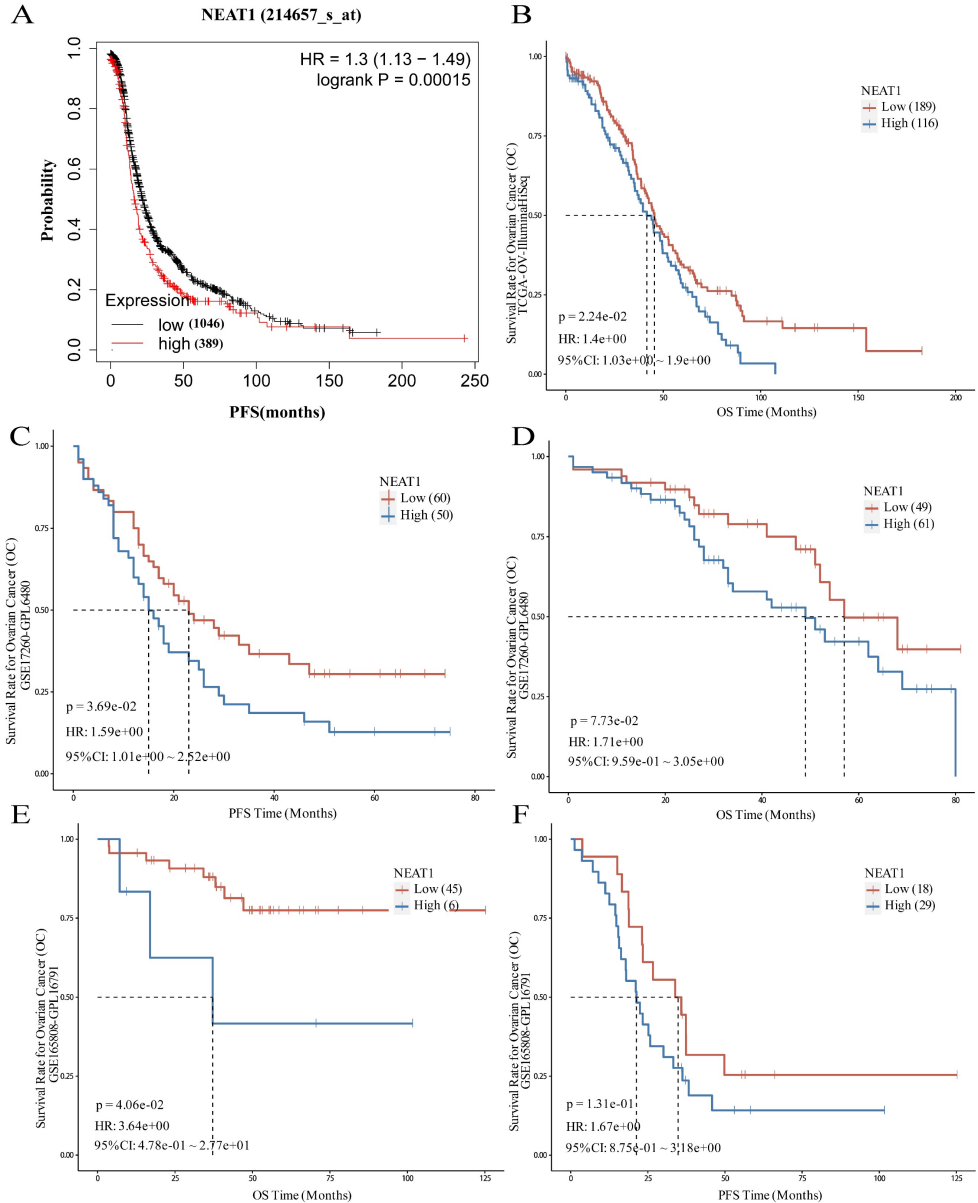
Prognostic role of NEAT1 in ovarian cancer. HR: Hazard Ratio, CI: Confidence Interval. **(A)** The Kaplan-Meier Plotter database analyzes the relationship between NEAT1 and ovarian cancer patients' progression-free survival (PFS). **(B)** The PanCanSurvPlot showed low expression of NEAT1 was positively correlated with overall survival (OS) in TCGA. **(C-D)** The PanCanSurvPlot showed that low expression of NEAT1 was positively correlated with poor OS and PFS of 110 HGSOC patients who received primary surgery and platinum/taxane-based chemotherapy in the GSE17260 dataset. **(E-F)** Kaplan-Meier curves for OS and PFS correlation with NEAT1 expression levels in 51 primary high-grade ovarian carcinoma patients treated with platinum-based chemotherapy in the GSE165808 dataset.

To evaluate the role of NEAT1 in patients with ovarian cancer, the prognostic value of NEAT1 was examined in the Kaplan-Meier Plotter databases using the default median method to define low or high groups. The prognostic value of NEAT1 in ovarian cancer patients was also evaluated in the Kaplan-Meier Plotter database, finding that increased expression of NEAT1 was related to poor prognosis (shorter PFS) of ovarian cancer patients (Figure 11A). The results obtained from the PanCanSurvPlot were consistent. Different groups were defined based on the optimal (maximally selected rank statistics) method to define whether low or high groups were consistent. We analyzed the ovarian cancer cohort from the Cancer Genome Atlas32 (TCGA), for which expression data were available for the polyadenylated NEAT1 transcript. In the TCGA-OV IlluminaHiSeq dataset, lower NEAT1 expression was positively correlated with overall survival (OS) (p = 0.0224, HR = 1.4) (Figure 11B).

The GSE17260 dataset 24 was also recruited in this study to evaluate the role of NEAT1. A high level of NEAT1 was correlated with poor PFS of patients with ovarian cancer (P = 0.0369, HR = 1.59) and poor OS (P = 0.073, HR = 1.71), respectively (Figure 11C-D).

### NEAT1 predicted chemotherapy response in OC

To evaluate whether NEAT1 expression levels correlate with response to chemotherapy, we chose a cohort of 51 primary HGSOC patients (GEO GSE165808) 25 because all patients included in the cohort had been exposed to first-line platinum-based chemotherapy, and expression levels of all human genes were available. Patients with high NEAT1 mRNA expression had worse prognosis. When we correlated the expression levels of NEAT1 with PFS, we found that the correlation was insignificant (P = 0.131; Figure 11F). however, the association between NEAT1 and OS, it showed significant correlation (P = 0.0406; Figure 11E).

We validated the prognostic value of NEAT1 expression using another HGSOC mRNA expression dataset (GSE17260) 24 downloaded from the GEO database. We selected the advanced serous ovarian cancer tissue data of 110 patients who received primary surgery and platinum/taxane-based chemotherapy. This analysis confirmed that NEAT1 expression is a good predictor of chemotherapy response in HGSOC (Figure 11C-D).

## Discussion

Epithelial ovarian cancer (EOC) has the highest fatality rate in gynecological cancer. PARP inhibitors could increase DNA damage and trigger DNA repair pathways. Although PARP inhibitors respond well to HR deficiency cancers, the issue of drug resistance has become a significant obstruction in clinical because of the restoration of HR 26. Thus, inhibiting homologous recombination repair increases PARPi sensitivity and improves the survival rate in serous ovarian cancers.

NEAT1, a component of the paraspeckles, is located on human chromosome 11 27. In a xenograft tumor mouse model, the PTX group or NEAT1 knockdown inhibited tumor growth, while combined sh-NEAT1 with PTX caused a more significant inhibition of tumor growth. Mechanistically, knockdown NEAT1-induced PTX sensitivity was mediated by miR-194/ZEB1 axis *in vivo* and *in vitro* 28. In another study, the knockdown of NEAT1 improved cisplatin response by increasing miR-770-5p and decreasing PARP1, demonstrating a novel treatment target for enhancing the efficacy of cisplatin in ovarian cancer cells 29. NEAT1 knockdown increased cisplatin/Taxol sensitivity in Triple-Negative Breast Cancer (TNBC) 30. Furthermore, the knockdown of NEAT1 could reverse the paclitaxel resistance in non-small cell lung cancer (NSCLC) by inducing apoptosis by increasing cleaved PARP and cleaved caspase-3 expression 31. However, there were no reports associated with PARPi sensitivity in ovarian cancer. Consistent with those studies above, we found that the knockdown of NEAT1 decreased cell viability in Olaparib-treated ovarian cancer cells. NEAT1 knockdown induced synthetic lethality with PARP inhibitors in human ovarian cancer cell lines (HeyA8 and SKOV3). Therefore, targeting NEAT1 is vital to enhance the sensitivity of tumor cells to DNA-damaging agents such as PARP inhibitors.

Pathways that participated in HR repair were significantly inhibited in NEAT1 KD cell lines detected by the Gene Set Enrichment Analysis (GSEA). In human myeloma cell lines AMO-1 and NCI-H929, RAD51B, RAD51D, p-CHK1, p-CHK2, RPA32 and BRCA1 protein expression reduced after silencing NEAT1 12. All proteins were involved in the DNA damage checkpoint pathway. ATR-mediated phosphorylation of checkpoint kinase CHK1 and replication protein RPA32 expression was inhibited in NEAT1 and NEAT1_2 knockdown U2OS cells exposed to HU 11.

Our previous research found a decrease in RAD51 levels via microarray assays after miR-506 overexpression in ovarian cancer cell lines and further demonstrated that miR-506 directly targeted RAD51 13. We predicted NEAT1 as a ceRNA of miR-506-3p through Starbase, LncTar, and LncBase databases. Therefore, we speculated whether NEAT1, as a ceRNA of miR-506-3p, could regulate DNA homologous recombination repair. Our data indicated that silencing NEAT1 could decrease homologous recombination gene expression through down-regulating FOXM1 and RAD51. In addition, several studies have found that RAD51 nuclear foci can be an alternative marker for HR and be used to forecast the response to PARPi 32. Furthermore, we found that knockout of NEAT1 decreased RAD51 foci formation, indicating the HR defect.

Silencing of both NEAT1_1 and NEAT1_2 in MCF-7 cells and U2OS cells caused an accumulation of DNA damage, apparent increases in γ-H2AX levels, and γ-H2AX foci formation. PARP inhibitors cause DNA damage and increase replication stress 11. Indeed, NEAT1 knockdown caused massive DNA damage and increased pH2AX foci 12. In line with these data, we found that knockout of NEAT1 increased γ-H2AX foci formation and protein expression of γ-H2AX, indicating increased DNA damage. We demonstrated that knocking NEAT1 inhibited PARP inhibitor-induced DSB repair and increased Olaparib-induced DNA damage.

Our previous research initially found that miR-506-3p could increase the sensitivity to chemotherapy and PARPi by regulating the RAD51-HR and EZH2/β-catenin pathway 14. Considering that knockdown NEAT1 inhibited the activity of the HR pathway and increased DNA damage, we evaluated whether knockdown NEAT1 could increase OC sensitivity to Olaparib by targeting RAD51-HR in OC cells. Further rescue experiments demonstrated that overexpression RAD51 rescued the inhibitory effects of NEAT1. These results indicated that ovarian cancer could benefit from NEAT1 targeting, as this approach could sensitize ovarian cancer to PARP inhibitors and other DNA-damaging agents. However, the expression and predictive roles of NEAT1 were assessed in ovarian cancer cell lines and a database of OC patients. As we all know, the *in vitro* cell experiments could not perfectly simulate the tumor microenvironment in patients. Therefore, further investigations *in vivo* and clinical patients could deeply explain the mechanisms of targeting NEAT1 increased PARPi sensitivity in the future.

Novel diagnostic technologies, including microarrays, RNA sequencing (RNA-seq), and qRT-PCR, have been used to quantify lncRNAs. In recent years, nanomedicine has made tremendous progress in delivering non-coding RNAs using nanoparticles. Some small molecule inhibitors, siRNAs, antisense oligonucleotides, and CRISPR-Cas9 have progressed, and indirect regulators of lncRNAs are also pointed out a new window in drug advancement. This therapeutic strategy might be rapidly amenable to the clinic. However, the reason for limiting NEAT1 as the target is that there is no proper NEAT1 inhibitor and proper carriers to deliver target drugs clinically. Further research remains to be done to remove the obstructions associated with *in vivo* delivery of lncRNAs.

In conclusion, NEAT1 was highly expressed in OC, and OC patients with NEAT1 high expression showed a poor prognosis, including OS and PFS. More importantly, NEAT1 could predict the response to chemotherapy in OC. Mechanistically, we showed that targeting NEAT1 increased the response to PARPi *in vitro* in serous ovarian cancer by directly influencing the RAD51-HR axis, further indicating the critical role of NEAT1 in improving OC chemotherapy sensitivity and providing promising treatment strategy for the application of NEAT1 in OC. Our results provided the theoretical foundation for clinical research aiming at combining PARP inhibitors associated with NEAT1 inhibitors to treat ovarian cancer patients.

## Supplementary Material

Supplementary figures.

## Abbreviations

PARPi: PARP inhibitor
HR: homologous recombination
TCGA: The Cancer Genome Atlas
ICGC: International Cancer Genome Consortium
OC: ovarian cancer
HRD: homologous recombination deficiency
DSBs: double-strand breaks
RPA: replication protein A
SCLC: small-cell lung cancer
NEAT1: nuclear paraspeckle assembly transcript 1
HMCLs: human myeloma cell lines
ceRNA: competing endogenous RNA
HGSOC: high-grade serous ovarian carcinoma
CCLE: Cancer Cell Line Encyclopedia
OS: overall survival
PFS: progression-free survival
GO: gene ontology
KEGG: kyoto encyclopedia of genes and genomes
FBS: fetal bovine serum
ASOs: antisense oligonucleotides
EOC: epithelial ovarian cancer
TNBC: triple-negative breast cancer
NSCLC: non-small cell lung cancer
GSEA: gene set enrichment analysis
