# Supplementary material for: Targeting NEAT1 Affects the Sensitivity to PARPi in Serous Ovarian Cancer by Regulating the Homologous Recombination Repair Pathway
Source: J Cancer. 2024 Jan 20;15(5):1397–413. doi: 10.7150/jca.91896 (PMC10861825; doi:10.7150/jca.91896)
Supplement: Supplementary file 1 — Supplementary figures. [file jcav15p1397s1.pdf]

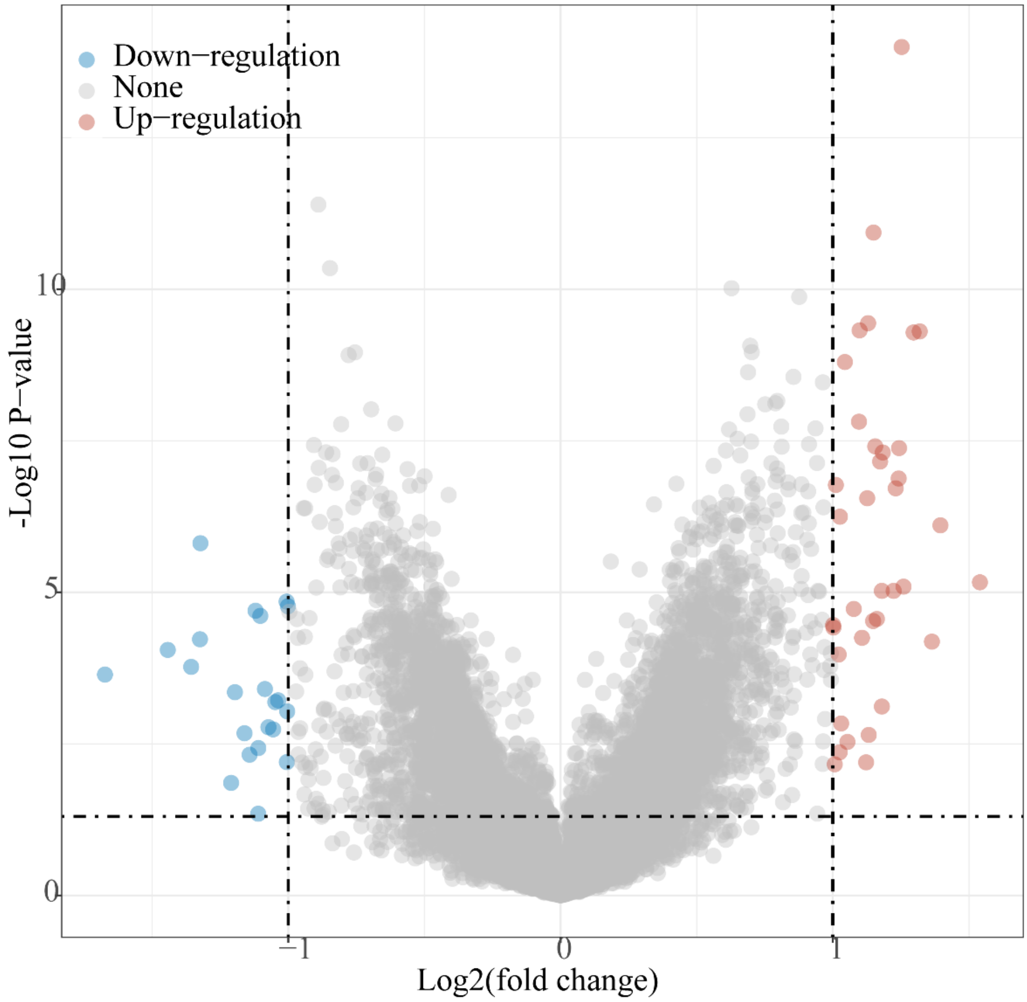

Supplementary Figure 1. The volcano plot was constructed using the fold change values and P-adjust. $P < 0.05$ was set as the cut-off criterion of significant difference. Red dots indicate upregulated genes; blue dots indicate downregulated genes; grey dots indicate not significant.

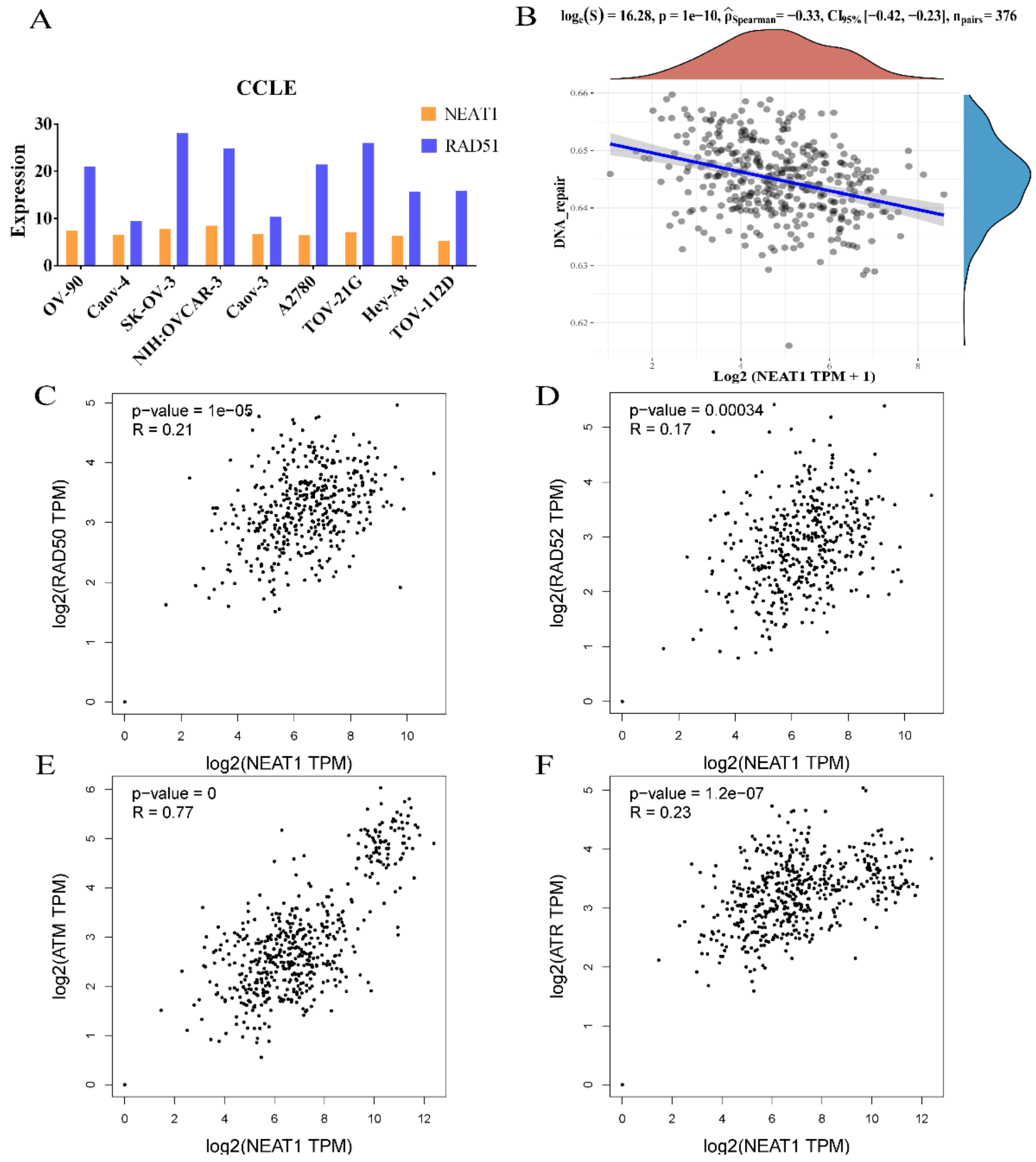

Supplementary Figure 2. The relationship between NEAT1 and HR repair pathway.

(A) CCLE databases showed the expression of NEAT1 and RAD51 in different ovarian cancer cell lines.

(B) The correlations between NEAT1 and DNA repair pathway score were analyzed with Spearman. The abscissa represents the distribution of the gene expression, and the ordinate represents the distribution of the pathway score. The density curve on the right represents the trend in the distribution of pathway immune score, the upper-density curve represents the trend in the distribution of the gene expression.

(C-F) The scatter plot showed the correlation between the expression of NEAT1 and HR-associated genes (RAD50, RAD52, ATM, ATR) by EPIC algorithm.

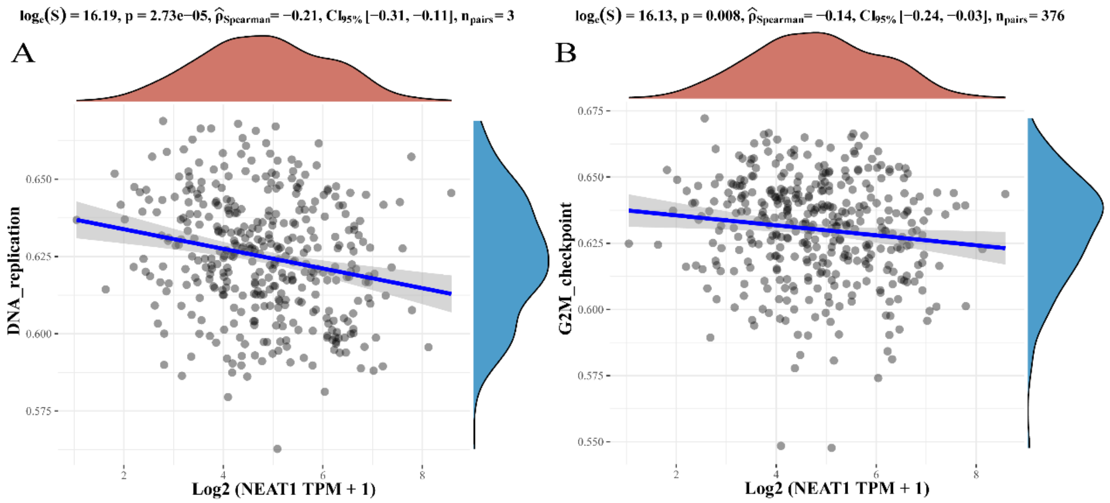

Supplementary Figure 3. The relationship between NEAT1 and DNA replication and G2-M checkpoint pathway.

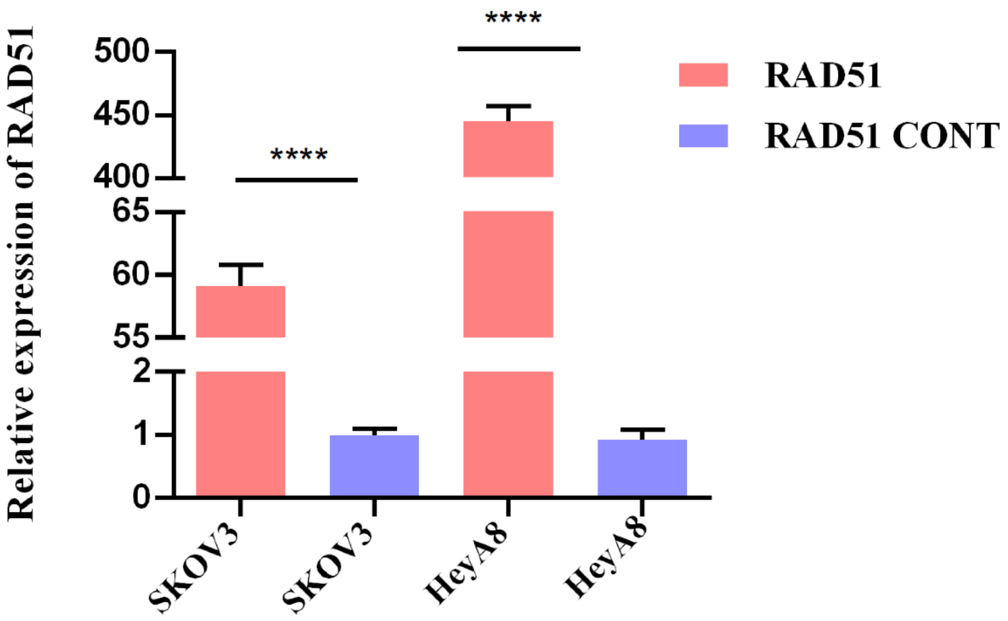

Supplementary Figure 4. qRT-PCR analyzed the RAD51 mRNA levels after transfecting the overexpression RAD51 plasmid or empty vector into HeyA8 and SKOV3 cells.

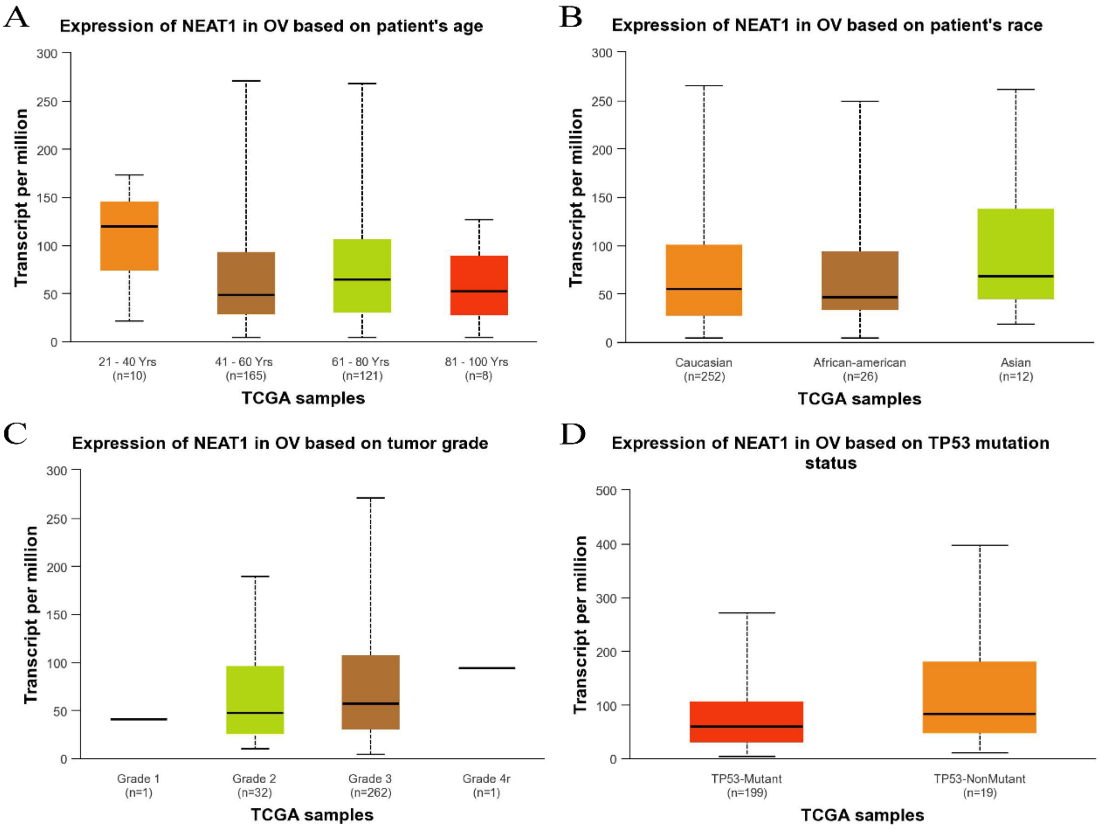

Supplementary Figure 5. Relationship between NEAT1 expression and clinicopathological features in OC. The correlation between NEAT1 expression and different clinical features, including age (A), race (B), tumor grade (C), and TP53 mutation status (D). Subtype descriptions: Grade 1: Well differentiated (low grade); Grade 2: Moderately differentiated (intermediate grade); Grade 3: Poorly differentiated (high grade); Grade 4: Undifferentiated (high grade).
